# 187. Characterizing Household Clustering of COVID-19 Cases in Fulton County, Georgia, June 2020–April 2021

**DOI:** 10.1093/ofid/ofab466.187

**Published:** 2021-12-04

**Authors:** Carol Liu, Sasha Smith, Allison Chamberlain, Neel Gandhi, Fazle N Khan, Steve Williams, Sarita Shah

**Affiliations:** 1 Emory University, Atlanta, Georgia; 2 Fulton County Board of Health, Atlanta, Georgia; 3 Rollins School of Public Health, Atlanta, Georgia; 4 Georgia Depat. of Public Health, Atlanta, Georgia; 5 Fulton County, Atlanta, Georgia

## Abstract

**Background:**

Households are important for SARS-CoV-2 transmission due to close proximity in enclosed living spaces over long durations. Using contact tracing, the secondary attack rate in households is estimated at 18-20%, yet no studies have examined COVID-19 clustering within households, an important measure to inform testing and prevention. We sought to quantify and characterize household clustering of COVID-19 cases in Fulton County, Georgia.

**Methods:**

We used state surveillance data to identify all PCR- or antigen-confirmed cases of COVID-19 in Fulton County. Clustered cases were defined as cases with matching street address, including unit number. Communal places (e.g., nursing homes, correctional facilities) were excluded, as were apartments missing unit number. Household clusters were defined as ≥2 COVID-19 cases at the same residential address with positive sample collection dates within 14 days of one another. We described proportion of COVID-19 cases that were clustered, stratified by age, sex, and race/ethnicity over time.

**Results:**

There were 60,614 COVID-19 cases with available address reported in Fulton County during 6/1/20–4/30/21. Of these, 25,149 (41.6%) had an address that matched at least one other case; 20,793 (34.3%) were from 8,582 household clusters with positive sample collection dates within 14 days (Fig 1). Majority of clusters had 2 individuals (N=6119, 71%), though some had ≥6 individuals (N=79, 0.9%). Clustering increased through January 2021 (Fig 2). Children were more likely to be in household clusters (Fig 4) and 15% of clusters had a child as first diagnosed case with increases since January 2021 (Fig 3). Consistently higher clustering was observed among Hispanic persons, with rising clustering among Asian persons (Fig 5).

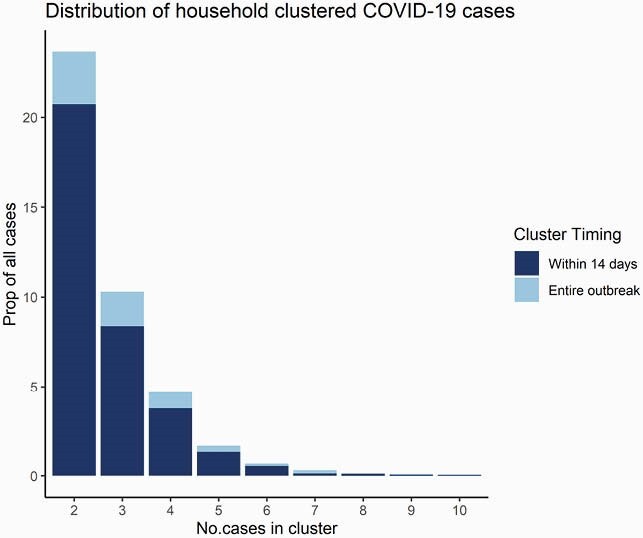

Figure 1. Distribution of household-clustered COVID-19 cases in Fulton county between June, 2020 and April 2021

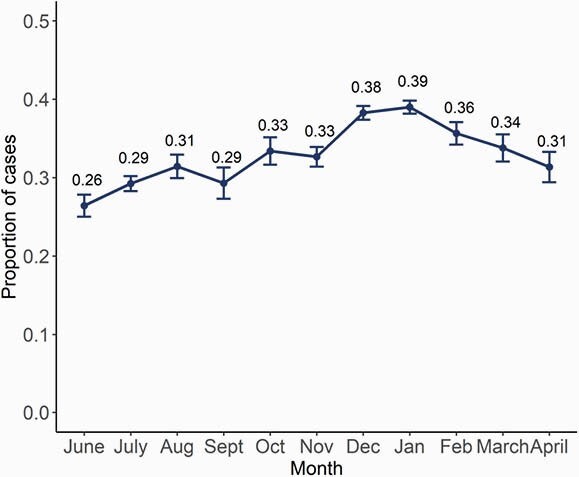

Figure 2. Proportion of COVID-19 cases that were part of a household cluster, Fulton County, June 2020–April 2021. Error bars denote 95% confidence interval around the point estimate.

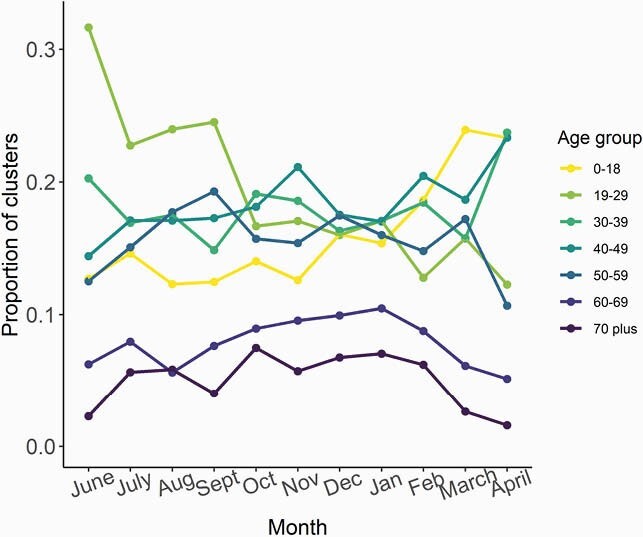

Figure 3. Age of first diagnosis among households with at least 2 cases diagnosed within 14 days

**Conclusion:**

One-third of COVID-19 cases in Fulton County were part of a household cluster. The higher proportion of children in household clusters likely reflects higher probability of living in a home with an adult caregiver. Higher household clustering among Hispanic and Asian persons, regardless of age, may reflect larger households (supported by census data) or increased exposures outside the house. Timely testing for household members to prevent ongoing transmission remains essential.

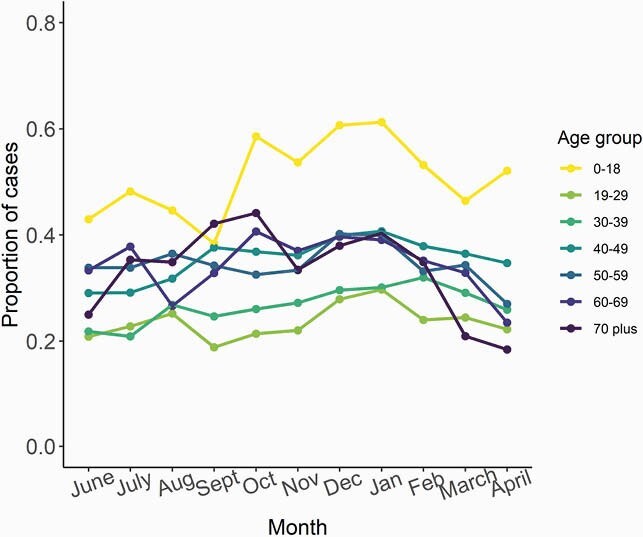

Figure 4. Proportion of COVID-19 cases that were part of a household cluster, by age – Fulton County, June 2020–March 2021

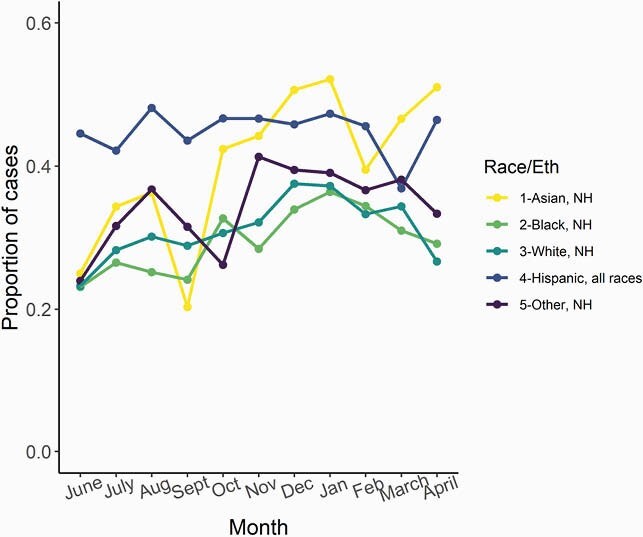

Proportion of COVID-19 cases that were part of a household cluster in Fulton County stratified by race/ethnicity over time

**Disclosures:**

**All Authors**: No reported disclosures

